# Dietary supplementation of soy germ phytoestrogens or estradiol improves spatial memory performance and increases gene expression of BDNF, TrkB receptor and synaptic factors in ovariectomized rats

**DOI:** 10.1186/1743-7075-7-75

**Published:** 2010-09-15

**Authors:** Meixia Pan, Zhuoneng Li, Victor Yeung, Ruo-Jun Xu

**Affiliations:** 1 Dept. of Nutrition, Guangdong Academy of Medical Sciences, Guangdong General Hospital, No.106, Zhongshan Er Road, Guangzhou 510080, China; 2 Food Safety Section, Wuhan Centres for Disease Prevention and Control, No.24 JiangHan Bei Road, Wuhan 430022, China; 3 School of Biological Sciences, The University of Hong Kong, Hong Kong SAR, China

## Abstract

**Background:**

Estrogen or phytoestrogens treatment has been suggested to improve cognitive function of the brain in postmenopausal women. However, there is lack of information on the mechanism of such treatment on the central nervous system. The present study aimed to determine the effects of estradiol and soy germ phytoestrogens on spatial memory performance in ovariectomized rats and to explore the underlying mechanisms affecting the central nervous system.

**Methods:**

Ovariectomized Sprague-Dawley rats were fed a basic diet supplemented with soy germ phytoestrogens (0.4 g/kg or 1.6 g/kg) or 17β-estradiol (0.15 g/kg) for 12 weeks. At the end of the experiment, animals were evaluated for their spatial learning and memory performance by the Morris Water Maze task. The expressions of brain-derived neurotrophic factor (BDNF) and synaptic formation proteins in the hippocampal tissue were estimated using RT-PCR and ELISA.

**Results:**

It was found that rats supplemented with soy germ phytoestrogens or estradiol performed significantly better in spatial memory acquisition and retention when compared to the rats fed on the control diet. Estradiol or the high dose of phytoestrogens treatment significantly increased BDNF concentration and the mRNA levels for BDNF and its TrkB receptors as well as the synaptic formation proteins, synaptophysin, spinophilin, synapsin 1 and PSD-95, in the hippocampal tissue of the experimental animals. It was also found that phytoestrogens, in contrast to estradiol, did not show any significant effect on the vaginal and uteri.

**Conclusion:**

Soy germ phytoestrogens, which may be a substitute of estradiol, improved spatial memory performance in ovariectomized rats without significant side-effects on the vaginal and uteri. The memory enhancement effect may relate to the increase in BDNF and the synaptic formation proteins expression in the hippocampus of the brain.

## Background

It has been reported in the literatures that estrogen supplement treatment improves memory acquisition and retention in ovariectomized rats [1] and postmenopausal women [2]. However, estrogen supplement increases the risk of developing uterine and breast cancer in postmenopausal women [3]. Phytoestrogens supplement has been considered to be a potential alternative treatment without server side effects on the breast and the uterus [4]. Phytoestrogens are a group of compounds with a diphenolic structure similar to that of natural and synthetic estrogens [5]. Phytoestrogens of all chemical groups are widely spread in fruits, vegetables, legumes, whole grains and soy products [6]. It was reported that soy diet rich of phytoestrogens improved working memory in the ovariectomized retired breeder rats [7]. However, there is a lack of understanding in the molecular mechanism of phytoestrogens effects on the brain. Brain-derived neurotrophic factor (BDNF) plays a crucial role in the brain; it regulates the survival, differentiation and phenotypic maintenance of various neuronal populations [8]. It has also been reported that the system of BDNF/tyrosine kinase receptors B (BDNF/TrkB) is expressed in the hippocampus region of the brain and it plays a crucial role in memory acquisition and retention [9]. BDNF improves the survival of hippocampal neurons and restores hippocampal neurogenesis [10,11]. Our preliminary study showed that estradiol and phytoestrogens, genestein and daidzein increased BDNF expression in fetal rat hippocampal neurons *in vitro *(data not shown). Thus, we suspect that the action of phytoestrogens or estradiol on the central nervous system, particularly on its learning and memory function, may be mediated by BDNF expression. The objective of the present study was to examine the effects of dietary supplementation of estradiol or phytoestrogens on the spatial reference memory behavior in overiectomized rats and its relation to BDNF and TrkB receptor expression in the hippocampal region of the brain. As synaptic formation in the hippocampus plays an important role in learning and memory function of the brain [12], the effects of dietary supplementation of estradiol or phytoestrogens on hippocampal gene expression of various synaptic formation proteins were also examined.

## Methods

### Animal experiment

The experimental protocol was approved by the animal ethic committee of the University of Hong Kong (approval code: 1072-05). Twenty-eight female Sprague-Dawley rats aged 3 months were obtained from the Laboratory Animal Unit of the University of Hong Kong. All animals received a surgical operation to remove both ovaries under general anaesthesia of intraperitoneal injection of ketamine (60 mg/kg, Sigma, USA) and xylazine (10 mg/kg, Sigma, USA). After eighteen days of recovery, the ovariectomized rats were randomly segregated into four treatment groups (n = 7). Animals in one group were maintained on the control diet (Table 1) for 12 weeks. Animals in the remaining three groups were maintained on the control diet supplemented with 0.15 g/kg 17β-estradiol, or supplemented with 0.4 g/kg or 1.6 g/kg soy germ phytoestrogens respectively. The phytoestrogens was a soy germ product (SoyLife 40%, ACATRIS, Netherlands, Batch No. 01 M/1910/4), containing 152 mg/g daidzein, 80 mg/g glycitein and 35 mg/g genistein.

**Table 1:** The composition of the control diet based on the formula of AIN-93G purified diet.

Ingredient	Concentration (g/kg)
Casein	200
L-cystine	3
Corn starch	397.486
Maltodextrin	132
Sucrose	100
Corn oil	70
Cellulose	50
Mineral-mix (AIN-93G-MX)	35
Viramin-mix (AIN-93G-VX)	10
Choline Bitartrate	2.5
t-Butylhydroquinone (TBHQ)	0.014

### Cognitive testing with Morris Water Maze

At the end of the feeding experiment all animals were evaluated for their spatial memory performance by Morris Water Maze (MWM) test. The swimming pool used for the test was 190 cm in diameter and 60 cm deep. The escape platform (100 cm^2^) was fixed in a permanent position 2 cm under the water surface during the course of the MWM training procedure. The quadrant housing the escape platform was defined as the target zone. The water in the pool was made opaque with coffee-mate powder to prevent the rats from seeing the platform, and the temperature of the water was maintained at 22-25°C. Spatial reference cues (arrow, star, circle, and rectangle) around the pool were remained constant during the test. For spatial learning acquisition test, the rats were trained in MWM for 5 consecutive days using 3-trial-per-day regime. The rats were placed into the pool facing the wall randomly from one of the three starting points located in the three quadrants except the quadrant with the platform. If the animals failed to find the platform by the maximum period of 120 seconds, they would be gently placed on the platform. At the end of each trial, the rats were allowed to rest on the platform for 30 s. The time (escape latency) and swimming distance to reach the platform were recorded by a video camera and analyzed using the computer software (Noldus). To assess spatial memory retention, a probe trial was performed 1 day after the last training trial, during which the platform was removed from the pool, while all other factors remained unchanged. Rats were allowed to swim for 90 s.

### Vaginal smear, uterus and brain isolation

Estrous status was observed using vaginal smear performed for 10 consecutive days from the first day of the 11^th ^week of the feeding experiment. Vaginal smears were obtained by flushing the rats' vagina with 0.2 ml 0.9% saline with a blunt-end tip. The resulting suspension was placed on a slide, covered with a cover slip and examined with a microscope.

After completion of the spatial memory tests, the animals were euthanized. The brains were rapidly removed and placed on ice. The hippocampus were then isolated, frozen in liquid nitrogen and stored at -80°C.

The uterus of each animal was removed and weighted, and then fixed in 10% buffered formalin for 48 h. The right side of the proximal region of each uterus was embedded in paraffin wax, and 5 μm cross tissue sections were stained with hematoxylin and eosin (H&E) for histological evaluation.

### BDNF extraction and assay

The extraction of BDNF from the hippocampal tissue was performed on ice and following the description of Szapacs [13]. In brief, the tissue was suspended in 5 volume of lysis buffer containing 137 mM NaCl, 20 mM Tris-HCl, 1% NP40, 10% glycerol, 1 mM PMSF, 0.5 mM sodium vanadate and protein inhibitor cocktail (Calbiochem, USA). The suspension was homogenized on ice for 20 s using a sonicater at power level 3 and pulses at 1 s. The homogenates were then centrifuged at 16000×g for 30 min at 4°C. The resulting supernatant was stored at -80°C for further analyses.

Mature BDNF was measured using a sensitive two-side ELISA kit (BDNF-Emax ImmunoAssay system, Promega) following the manufacturer's instructions. In brief, 96-well ELISA plates were coated with 100 μl/well of anti-BDNF monoclonal antibody and incubated overnight at 4°C. Following wash with the washing buffer containing 0.05% (v/v) Tween 20, 20 mM Tris-HCl, and 150 mM NaCl, pH 7.6, the plate was incubated for 1 h with 200 μl/well of block & sample buffer to prevent non-specific binding. The plate was washed again and 100 μl/well of samples or standard (0-500 pg rhBDNF/ml) was added to the plate in duplicates followed by incubation with shaking for 2 h. After washing, 100 μl/well of anti-human BDNF antibody (1 μg/ml) was added followed by 2 h incubation with shaking. After washing again, 100 μl/well of Anti-IgY HRP was added followed by 1 h incubation with shaking. After the last washing, 100 μl/well of tetramethylbenzidine solution was added followed by 10 min incubation with shaking. The enzymatic reaction was stopped by addition of 100 μl/well of 1N HCl. The absorbance of the reaction product was measured within 30 min at 450 nm using a micro-plate reader. The concentration of BDNF in the samples was calculated from the rhBDNF standard curve by linear regression analysis performed on each micro-plate, and the BDNF were expressed as pg of BDNF per mg protein. The total BDNF in the sample was measured after the transient acidification treatment of the sample below pH3.

### Quantification of mRNA expression for BDNF and its receptor TrkB and various synaptic formation proteins

Reverse transcription polymerase chain reaction (RT-PCR) was used to evaluate the mRNA levels for BDNF (NM012513), TrkB (NM012731), synaptotagmin 1 (NM001033680), synaptophysin (NM012664), synapsin 1 (X04655), PSD-95 (N96853) and spinophilin (AF016252). The mRNA for GADPH (NM017008) was used as an internal control. Primers specific to target genes were designed from public sequences using Primer 3 software http://fokker.wi.mit.edu/primer3/input.htm. Sequences of PCR primers were shown in Table 2.

**Table 2:** Sequences of PCR primers and conditions of PCR amplification of cDNA.

						Conditions of PCR amplification of cDNA			
						
Primer name	Sequences	Products (bps)	Tm	length	OD's	Denaturation	Annealing	Extension	Cycles
GAPDH forward	gggtgtgaaccacgagaaat	481	47	20	11	94°C for 30 s	55°C for 30 s	68°C for 35 s	33
GAPDH reverse	ggaagaatgggagttgctgt		47	20	10.1				
BDNF forward	tgtgacagtattagcgagtgggt	219	59.1	23	5	94°C for 30 s	50°C for 30 s	68°C for 15 s	40
BDNF reverse	cgattgggtagttcggcatt		60	20	5				
TrkB forward	cttatgcttgctggtcttgg	503	47	20	10.4	94°C for 30 s	59°C for 60 s	72°C for 35 s	38
TrkB reverse	gggtattcttgctgctctca		47	20	9.4				
Synaptophysin forward	catcttcgcctttgctacg	508	46	19	10.8	94°C for 30 s	55°C for 30 s	68°C for 35 s	40
Synaptophysin reverse	cactgaggtgttgagtcctga		49	21	8.8				
synaptotagmin 1 forward	gttgcggtccttttagtcgt	496	47	20	9.3	94°C for 30 s	55°C for 30 s	68°C for 35 s	33
synaptotagmin 1 reverse	agtcatacacagccatcacca		47	21	9.6				
synapsin 1 forward	agcagcacaacataccctgtag	459	50	22	12.3	94°C for 30 s	52°C for 30 s	68°C for 35 s	40
synapsin 1 reverse	gaccacaagttccacgatga		47	20	7.7				
PSD-95 forward	gccctgtttgattacgaca	492	44	19	8.2	94°C for 30 s	55°C for 30 s	68°C for 35 s	40
PSD-95 reverse	gaacttgtgtgcctggatgt		47	20	8.7				
spinophilin forward	gaggaaagtggggagtctga	510	48	20	8.2	94°C for 30 s	58°C for 30 s	72°C for 35 s	37
spinophilin reverse	ctcattgcgtcggtcatagt		47	20	7.8				

Total RNA was extracted using AllPrep™ DNA/RNA/Protein Mini Kit (Qiagen, USA). The concentration and purity of RNA were measured by the optical density at 260 and 280 nm using spectrophotometer (Bio-Rad). Reverse transcription (RT) reactions were performed in duplicates with SuperScript™ III First-strand Synthesis SuperMix (Invitragen, USA). PCR was performed with 1 μl of cDNA in 25 μl reaction mixture containing 2.5 μl of 10× AccuPrimer™ PCR Buffer II, 0.5 μl primer mix (10 μM each), 0.5 μl AccuPrimer™ Taq DNA Polymerase (Invitrogen, cat.No.12339-016, USA). The conditions of PCR amplification of cDNA were shown in Table 2. Finally, 5 μl of the PCR products was resolved by 1% agarose gel electrophoresis, stained with SYBR^® ^Safe DNA gel stain (Invitrogen, USA) and visualized under UV light. The density of the PCR products was analyzed by Quantity One software (Bio-Rad, USA). Quantity of the expressed BDNF mRNA was analyzed based on a gray value, and expressed as the ratio of the sample density to GAPDH density amplified from an identical RNA sample.

### Statistical analysis

Data are presented as the mean ± standard error of the mean (SEM). All data were evaluated for equality of variance before statistical analysis. Statistical analysis of experimental data was carried out using software SPSS v15.0 (USA). Statistical differences were determined by one-way or two-way ANOVA followed by Student-Newman-Keuls post hoc test. Differences were considered significant when p < 0.05.

## Results

### Body weight gain

There was no difference in the initial body weights among the four groups of animals. During the experimental period, rats in the control group and those in the group supplemented with low dose of phytoestrogens gained body weight steadily and followed the similar growth pattern. Rats in the group supplemented with high dose of soy germ phytoestrogens gained much less weight, while rat in the group supplemented with estradiol gained nearly no weight during the experimental period. By the end of the feeding experiment rats in the control group and in the groups supplemented with low dose of phytoestrogens, or high dose of phytoestrogens or estradiol treatment gained (96.28 ± 13.63)g, (89.15 ± 11.9)g, (48.67 ± 14.13)g and (2.10 ± 11.38)g of body weight, respectively.

### Uterine weight uterine morphologic characteristics and vaginal smear

The average weight of the uteri from the rats treated with estradiol was (0.56 ± 0.04)g, which was significantly greater than the weight from the control animals ((0.10 ± 0.01)g, p < 0.05). The average uterus weights of rats received the low or high dose of phytoestrogens treatment were (0.12 ± 0.01)g and (0.14 ± 0.01)g respectively, and they did not significantly differ from that of the controls.

**Figure 1:**
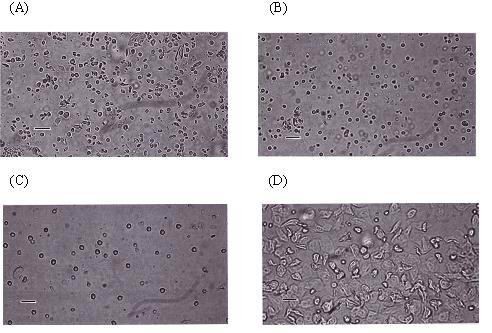
**Representative photomicrographs of vaginal smears**. The four groups of smears were performed in rats fed on the control diet (A), and on the diet supplemented with 0.4 g/kg phytoestrogens (B), 1.6 g/kg phytoestrogens (C) or 0.15 g/kg 17βestradiol (D). The length of the scale bar equals 250 um.

Representative vaginal smears of experimental animals were shown in Figure 1. The smear examination for the consecutive 10 days showed no estrus cycle in rats on the control diet or on the diet supplemented with low or high dose of phytoestrogens. The vaginal smear of these animals showed mainly leukocytes and a few irregularly shaped cornified epithelial cells. In contrast, the smear from the animals supplemented with estradiol showed preponderance of large, irregularly noncornified epithelial cells.

**Figure 2:**
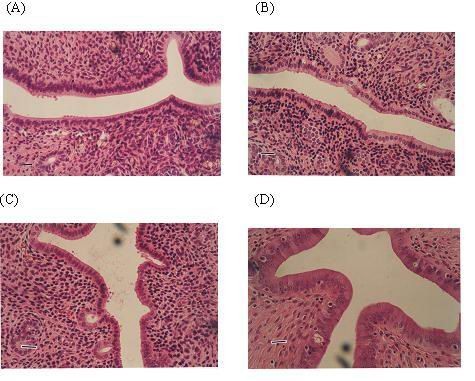
**Representative photomicrographs of uteri**. The four groups of photomicrographs come from rats fed on the control diet (A), and on the diet supplemented with 0.4 g/kg phytoestrogens (B), 1.6 g/kg phytoestrogens (C) or 0.15 g/kg 17βestradiol (D). The length of the scale bar equals 250 um.

The uterine histological characteristics of experimental animals were shown in Figure 2. In the control rats, the uterus appeared atrophic. The endometrium was composed of cuboidal inactive cells, and the connective tissue showed unorganized round nuclei. No mitotic activity was detected in epithelial cells. Similar morphologic characteristics were observed in the uterus of rats receiving low dose of phytoestrogens. In rats receiving high dose of phytoestrogens, endometrial cells of the uterus were stimulated but no pathologic signs were detected. However, in rats receiving estradiol treatment, endometrial mitotic activity was found, and all uterine structures were hypertrophic and hyperplastic.

### Behavioural Performance

**Figure 3:**
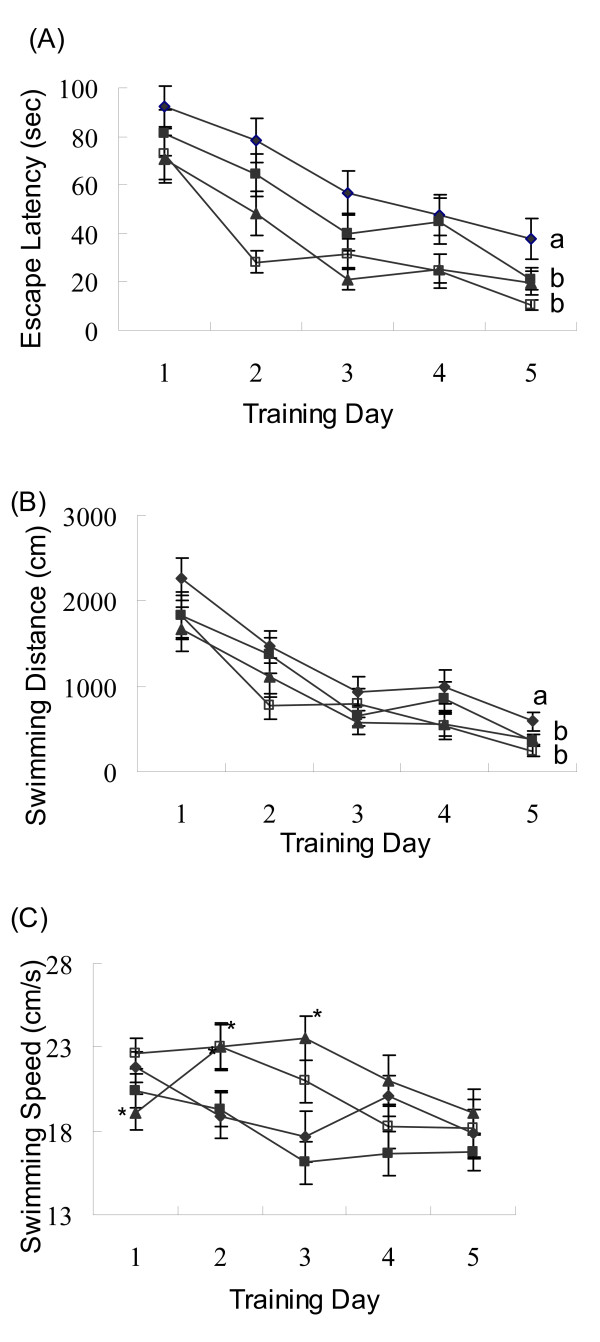
**Escape latency (A), swimming distance (B) and swimming speed (C) over the training period**. The animals were fed on the control diet (black diamond), or the control diet supplemented with 0.4 g/kg phytoestrogens (black square), 1.6 g/kg phytoestrogens (black triangle), or 0.15 g/kg 17βestradiol (white square) for 12 weeks. At the end of the experiment all animals were evaluated for their spatial memory performance with the Morris Water Maze task with a regime of 3 trials per day for 5 consecutive days. The data were presented as the means with the standard error bars (n = 7). Two-way ANOVA analysis showed significant differences in escape latency between different training days (F = 40.47, p < 0.001) and among treatment groups (F = 5.329, p < 0.01) with a significant interaction between the training time and the treatment (F = 2.558, p < 0.05). The analysis also showed significant differences in swimming distance between training days (F = 45.942, p < 0.001) and among treatment groups (F = 3.008, p < 0.05) with a significant interaction between the training time and the treatment (F = 3.063, p < 0.05). a, b: The mean values labelled with different letters differed significantly (p < 0.05). Significant differences from the mean values of the control on the same training day was indicated by * (p < 0.05).

To assess spatial learning acquisition, animals were trained with 3 trials per day for 5 consecutive days on the MWM task. The differences in escape latency (time to find the platform) and swimming distance of each training day among the four treatment groups were analyzed by two-way repeated measures ANOVA. On the first day of training, no difference was found in escape latency among the four groups. The escape latency gradually declined over the training period for all groups (Figure 3), indicating a gradual spatial memory acquisition in all experimental animals. Statistical analysis of two-way ANOVA (4 groups × 5 days) with repeated measures showed significant differences between the days of training (F = 40.47, p < 0.001) and among the treatment groups (F = 5.329, p = 0.002). There was also a significant interaction between the days of training and the treatments (F = 2.558, p = 0.046). On the 5^th ^day of training the escape latency of rats received the high dose of phytoestrogens or estradiol treatment was significantly shorter than that of the controls (p < 0.05, Figure 3).

A similar trend was observed in the swimming distance taken by rats to locate the platform. The distance reduced gradually for all animals over the training period (Figure 3). Statistical analysis of two-way ANOVA (4 groups × 5 days) with repeated measures showed significant differences between the days of training (F = 45.942, p < 0.001), and among the treatment groups (F = 3.008, p = 0.036) with a significant interaction between the training time and the treatment (F = 3.063, p = 0.034). On the 5^th ^day of training the swimming distance of rats received the high dose of phytoestrogens or estradiol treatment was significantly shorter than that of the controls (p < 0.05, Figure 3).

There was no significantly difference in the swimming speed among the treatment groups at the end of 5 day training (Figure 3). Statistical analysis showed that the swimming speed was negatively correlated with the body weight gains during the 12 weeks of feeding experiment (r = -0.230, p < 0.001).

**Figure 4:**
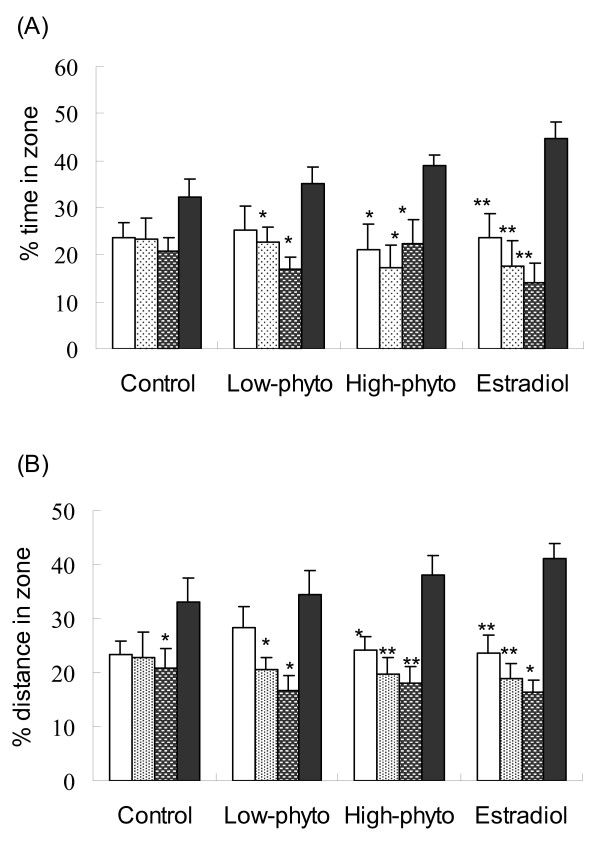
**Relative time spent and distance travelled in each of the quadrants in the probe test**. (A) Relative time spent, (B) relative distance. The four quadrants of the swimming pool were the target quadrant (dark grey), the quadrant clockwise adjacent to the target quadrant (white), the quadrant anticlockwise adjacent to the target quadrant (grey spotted), and the quadrant opposite to the target quadrant (white spotted). The experimental animals were fed for 12 weeks on the control diet (Control), or diet supplemented with 0.4 g/kg phytoestrogens (Low-phyto), 1.6 g/kg phytoestrogens (High-phyto) or 0.15 g/kg 17β-estradiol (Estradiol). The probe test was performed 1 day after the last training trial. During the probe test the platform was removed from the pool while all other conditions remained the same as in the training trails. Values are means with their standard errors represented by vertical bars (n = 7). Significant differences from the mean values of the target quadrant in each group were indicated by *(p < 0.05); ** (p < 0.001).

The results of the probe tests are presented in Figure 4. All animals showed a trend of spending more time in the target quadrant of the swimming pool. For animals received phytoestrogens or estradiol treatment, the relative time spent and distance travelled in the target quadrant were significantly greater than those in other quadrants (p < 0.05).

### Effects of estradiol and phytoestrogens treatment on BDNF and its TrkB receptor gene expression and expression of genes of synaptic formation proteins

**Figure 5:**
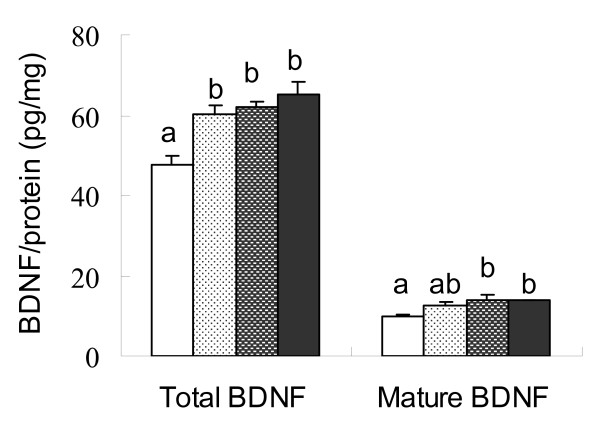
**The levels of total and mature BDNF in the hippocampal tissue of ovariectomized rats**. The levels (Mean ± SEM, n = 7) of total and mature BDNF in the hippocampal tissue of ovariectomized rats fed on the control diet (white), or the control diet supplemented with 0.4 g/kg phytoestrogens (white spotted), 1.6 g/kg phytoestrogens (grey spotted), or 0.15 g/kg 17β-estradiol (dark grey). The mean values labelled with different letters differed significantly (p < 0.05).

In the hippocampal tissue, BDNF was detected by a specific ELISA assay. The levels of both total BDNF and its mature form were significantly higher in animals received phytoestrogens or estradiol treatment when compared with that in control animals (F = 5.162, p < 0.05; F = 10.551, p < 0.05; Figure 5). The mature BDNF contributed about 20-22% of the total BDNF, and there was no significant difference among the four treatment groups in the conversion of pro-BDNF to mature BDNF.

**Figure 6:**
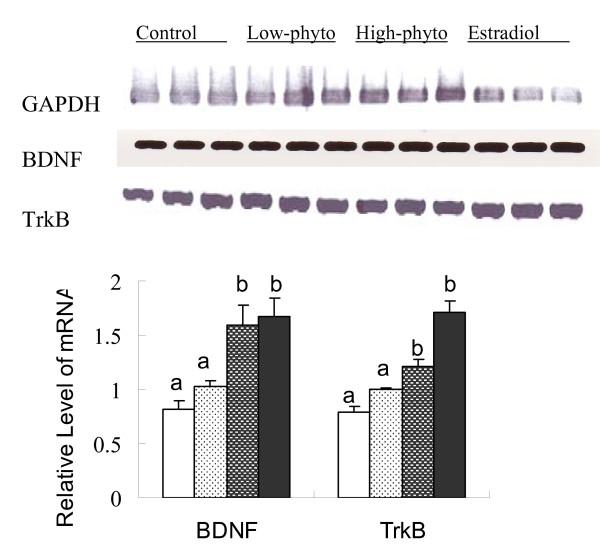
**The gene expression of BDNF and TrkB in the hippocampal tissue of ovariectomized rats**. The gene expression of BDNF and TrkB in the hippocampal tissue of ovariectomized rats fed on the control diet (white), or the control diet supplemented with 0.4 g/kg phytoestrogens (white spotted), 1.6 g/kg phytoestrogens (grey spotted), or 0.15 g/kg 17βestradiol (dark grey). The upper panel showed the PCR image and the lower panel presented the levels of BDNF and TrkB mRNA expressed as the ratio to the internal control of GADPH mRNA. The mean values labelled with different letters differed significantly (p < 0.05).

In accordance with the significant effects on BDNF levels, phytoestrogens or estradiol treatment increased BDNF gene expression (Figure 6). Compared with that of control animals, the BDNF mRNA level was significantly greater in the hippocampal tissue of the animals treated with estradiol or high dose of soy germ phytoestrogens (F = 3.469, p < 0.05). The level of BDNF mRNA was greater in the hippocampal tissue of the animals received low dose of phytoestrogens, although not significant, than that of control animals (Figure 6).

The mRNA level of TrkB, the primary receptor of BDNF, was also significantly greater in the hippocampal tissue of animals received estradiol or high dose of phytoestrogens treatment compared with that of the control animals (F = 3.244, p < 0.05).

**Figure 7:**
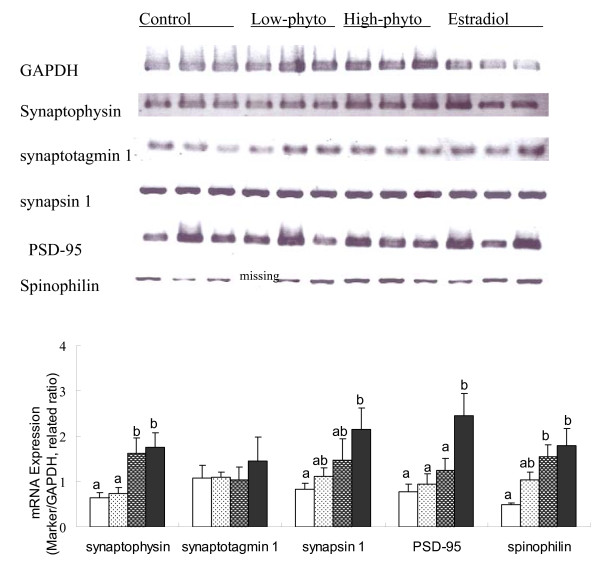
**The gene expression of sypnatic formation proteins in the hippocampal tissue of ovariectomized rats**. The gene expression of sypnatic formation proteins in the hippocampal tissue of ovariectomized rats fed on the control diet (white), or the control diet supplemented with 0.4 g/kg phytoestrogens (white spotted), 1.6 g/kg phytoestrogens (grey spotted), or 0.15 g/kg 17βestradiol (dark grey). The upper panel showed the PCR image and the lower panel presented the levels of mRNA of sypnatic formation proteins expressed as the ratio to the internal control of GADPH mRNA. The mean values labelled with different letters differed significantly (p < 0.05).

Synaptic formation plays an important role in learning and memory function of the brain. Figure 7 presents the relative expression levels of genes of various proteins related to synaptic formation in the hippocampal tissue of the experimental animals. The data showed that the mRNA levels of synaptophysin, synapsin 1 and spinophilin were significantly increased in rats received estradiol or high dose of soy germ phytoestrogens treatment when compared with that of the control animals (F = 3.557, p < 0.05; F = 3.453, p < 0.05; F = 3.363, p < 0.05). The mRNA level of PSD-95 was significantly increased in rats received estradiol treatment compared with that of the control animals (F = 3.284, p < 0.05). No difference in mRNA level of synaptotagmin 1 was observed among the treatment groups.

## Discussion

It has been reported that estrogen replacement therapy improves learning and memory function of the brain in ovariectomized aged rats [1] and postmenopause women [2]. However, estrogen treatment often has severe side effects and may increases the risk of uterine or breast cancer [3]. There have been wide interests in searching for alternative compounds, and phytoestrogens have been considered as potential candidates.

To evaluate the effect of estradiol and soy germ phytoestrogens on memory function, the spatial learning acquisition and memory retention of rats were tested using the Morris water maze (MWM) [14]. This task is based upon the premise that animals have evolved an optimal strategy to explore their environment and escape from the water with a minimum amount of effort, i.e., swimming the shortest distance possible. For spatial learning acquisition test, the time (escape latency) and swimming distance to reach the platform were recorded for each rat. To assess spatial memory retention, a probe trial was performed, during which the platform was removed from the pool, and the percentage of time spent in each quadrant was calculated and their swim paths were recorded by a video tracking system. The present study demonstrated that soy germ phytoestrogens, as well as estradiol, improved spatial learning and memory in ovariectomized rats. It was observed that, when compared to the animals fed on the control diet, rats fed on the diet supplemented with 1.6 g/kg soy germ phytoestrogens or 0.15 g/kg estradiol spent significantly shorter time to find the hidden platform (escape latency) during the Morris water maze training. Although the reduction in escape latency may partially result from the improved swimming speed, the same animals took significant shorter swimming distance to find the hidden platform than did the control animals (Figure 3). It was further showed that ovariectomized rats received the phytoestrogens or estradiol treatment had stronger spatial bias in the probe test than the controls (Figure 4). These findings suggest that dietary supplementation of phytoestrogens or estradiol improved memory acquisition and retention in ovariectomized rats. Similar findings have also been reported in the literature. Xu et al [15] reported that estradiol or genistein treatment given by subcutaneous injection reduced the escape latency of ovariectomized rats in a behavioral test. Estradiol or soy phytoestrogens treatment enhanced hippocampal-dependent spatial working memory in female mice [16] and ovariectomized retired breeder rats [17]. In postmenopausal women, dietary supplementation of soya isoflavones for 12 weeks significantly improved cognitive functions of the brain including learning rule reversals and planning task [18].

In contrast to the estradiol treatment, dietary supplementation of phytoestrogens showed no significant effect on the vaginal and uteri. Vaginal smear showed an estrus status in animals treated with estradiol but not in animals treated with control diet or phytoestrogens (Figure 1). We also found that estradiol, but not phytoestrogens, significantly increased the weight of uterus and stimulated cell proliferation in the uterus endometrium (Figure 2). Our data was supported by the report that daily treatment of genistein at 500 mg/kg(body weight) had no estrogenic effect in the uterus or the mammary gland in rats [19]. These findings indicate that dietary supplementation with phytoestrogens may have the benefit of improving cognitive function of the brain but without the severe side effect on the reproductive tract.

Although the findings of the present study and various earlier reports indicated that phytoestrogens or estradiol treatment has a beneficial effect on the brain cognitive function, how these compounds act on the brain is not clear. Consistent with Simpkins' [20] and Pan's [7] reports, we found that estradiol or phytoestrogens treatment significantly increased the levels of BDNF, especially mature BDNF, in the hippocampus, the known learning and memory centre of the brain. It was further showed that estradiol or phytoestrogens treatment significantly increased the mRNA levels for BDNF and its receptor TrkB in the hippocampus (Figure 6). BDNF is a member of the neurotrophin gene family which plays a crucial role in survival, differentiation, phenotypic maintenance, and in the selective vulnerability of various neuronal populations within the normal and diseased brain [8]. The postsynaptic BDNF-TrkB pathway is crucial for regulation of excitatory synaptic transmission and long-term potentiation (LTP) induction, which is an important synaptic connection model of memory formation [21]. This property implicates BDNF in the process of learning and memory [22,23]. Moreover, neurotrophins initially synthesized as precursors (proneurotrophins), they are cleaved to produce mature proteins, which promote neuronal survival and enhance synaptic plasticity by activating Trk receptor tyrosine kinases. Recent studies indicate that proneurotrophins serve as signalling molecules by interacting with the p75 neurotrophin receptor (p75NTR) which often has biological effects that oppose those of mature neurotrophins. Therefore, the proteolytic cleavage of proneurotrophins represents a mechanism that controls the direction of action of neurotrophins[24]. Although Murphy et al [25] found estrogen treatment temporally reduced BDNF in hippocampal cultures within 24 h of exposure, estrogen and/or phytoestrogens finally increased BDNF expression after certain period of culture. Indeed, it has been evidenced that estradiol regulated neurotrophins expression including BDNF [20,26-30]. Our study indicated that to observe the effect of phytoestrogens treatment, an effective period should be consider in the studies of both in vivo and in vitro. On the other hand, phytoestrogens might show different performance in vivo base on the gonadal hormones states. For the female, especially the peri-menopause with estrogen reducing, phytoestrogens will play a beneficial or substitute effect.

Synaptic formation plays an important role in the cognitive function of the brain. Synaptic loss is considered to be a reliable index of impaired cognition in dementia [31]. The present study demonstrated that estradiol or phytoestrogens treatment significantly increased the expression of genes of various proteins related to synaptic formation in the hippocampus. Dietary supplement of 0.15 g/kg estradiol or 1.6 g/kg phytoestrogens significantly increased the mRNA levels of synaptophysin, synapsin 1, PSD-95 and spinophilin in the hippocampus tissue (Figure 7). Synaptophysin, synaptotagmin 1 and synapsin 1 are belong to the presynaptic vesicle proteins which play an important role in synaptic plasticity and cognitive function [32]. Loss of the synaptophysin in hippocampus correlates with cognitive decline in Alzheimer's disease [33]. PSD-95 and spinophilin belongs to postsynaptic proteins involved in synapse stabilization and plasticity [34]. It is suspected that increased expression of genes of synaptic proteins may be partially responsible for the improved learning and memory performance following dietary supplementation of estradiol or phytestrogens in ovariectomized rats.

## Conclusions

In summary, the present study showed that phytoestrogens or estradiol treatment improved spatial memory acquisition and retention in ovariectomized rats. Unlike estradiol, phytoestrogens had no significant effect on the reproductive system. These finding suggest that phytoestrogens may be used in postmenopause women to improve cognitive function of the brain without the severe risk of developing uterus or breast cancer. The present study further showed that the increased gene expression for BDNF and its receptor TrkB and for various proteins related to synaptic formation in the hippocampus may be partially responsible for the improved spatial learning and memory performance in ovariectomized rats following dietary supplementation of estradiol or phytoestrogens.

## List of abbreviations

*BDNF*: brain-derived neurotrophic factor; *p75NTR*: p75 neurotrophin receptor; *MWM*: Morris water maze; *TrkB*: tyrosine kinase receptors B; *LTP*: long-term potentiation; *PSD-95*: postsynaptic density protein 95.

## Competing interests

The authors declare that they have no competing interests.

## Authors' contributions

MP participated in the design of the study, animal feeding, behaviours testing, and sample collection, gene and protein expression measurements, statistical analysis and drafting of the paper. ZL participated in the animal feeding, behaviours testing and sample collection. VY participated in the perform behaviours testing and management of molecular studies. RJX conceived the study, and participated in its design, interpretation and coordination and helped to draft the manuscript. All authors read and approved the final manuscript.
